# Hybrid delivery of cluster-set resistance training for individuals previously treated for lung cancer: the results of a single-arm feasibility trial

**DOI:** 10.1186/s40814-023-01405-z

**Published:** 2023-10-17

**Authors:** C. M. Fairman, O. L. Owens, K. L. Kendall, J. Steele, A. R. Schumpp, C. Latella, M. T. Jones, L. Marcotte, J. M. Dawson, C. M. J. Peddle-McIntyre, K. K. McDonnell

**Affiliations:** 1 Department of Exercise Science, University of South Carolina, Columbia, USA; 2 College of Social Work, University of South Carolina, Columbia, USA; 3 Centre for Human Performance, School of Medical and Health Sciences, Edith Cowan University, Joondalup, Australia; 4 Faculty of Sport, Health, and Social Science, Solent University, Southampton, UK; 5 Department of Kinesiology, The University of Alabama, Tuscaloosa, USA; 6 Faculty of Medicine, University of British Columbia, Vancouver, BC Canada; 7 Exercise Medicine Research Institute, School of Medical and Health Science, Edith Cowan University, Joondalup, Australia; 8 College of Nursing, University of South Carolina, Columbia, USA

**Keywords:** Resistance training, Lung cancer, Cluster sets, Dyspnea, Fatigue, Physical fitness, Quality of life

## Abstract

**Background:**

Individuals with non-small cell lung cancer (NSCLC) are burdened by long-lasting symptoms (e.g., dyspnea and fatigue) post-treatment. These symptoms often reduce physical activity levels and increase the risk of functional decline. Though we have previously proposed cluster-set resistance training to mitigate symptom burden in lung cancer, there is currently no data on the feasibility or acceptability of this mode of exercise in cancer. Therefore, the purpose of this study was to investigate the feasibility and acceptability of a hybrid-delivery home-based cluster-set resistance training program in individuals with NSCLC stages I–III (i.e., early stage).

**Methods:**

This study aimed to recruit individuals with NSCLC stages I–III post-treatment to participate in 8 weeks of home-based resistance training, 3 days per week. The program included supervised sessions in the participants’ homes and virtual supervision via videoconferencing. The primary outcome measure of feasibility was evaluated through recruitment, retention, and intervention fidelity (i.e., proportion of exercise completed, relative to what was prescribed). Intervention acceptability (i.e., ease and quality of virtual delivery, level of difficulty, and home-based approach) was assessed using a 4-point Likert-type scale from “strongly disagree” to “strongly agree”.

**Results:**

Fourteen participants were recruited over a 6-month period, with 11 completing the intervention (2 withdrew due to unrelated illness, 1 withdrew due to requiring active treatment), yielding a retention rate of 79%. Characteristics of the participants who completed the intervention (*n* = 11) were as follows: mean age: 71 ± 10 years, mean BMI: 29.1 ± 6.5, and average time since diagnosis was 62 ± 51 months. Of completers, 27% were male, and 36% were Black; 10 were stage I (91%), and one was stage II (9%). Mean session attendance was 86.4 ± 9.5%. Mean intervention fidelity was 83.1 ± 13.1%. With regard to acceptability,  > 90% of participants positively rated all aspects of the intervention delivery. No adverse events related to exercise were recorded.

**Conclusions:**

The hybrid delivery of a home-based resistance exercise program for individuals previously treated for early-stage NSCLC was found to be safe and feasible. Adaptations to the program for future interventions are required, particularly surrounding resistance exercise programming, and intervention delivery with home visits.

**Trial registration:**

ClinicalTrials.gov: NCT05014035. Registered January 20, 2021.

**Supplementary Information:**

The online version contains supplementary material available at 10.1186/s40814-023-01405-z.

## Key messages regarding feasibility

- What uncertainties existed regarding the feasibility? There were uncertainties whether individuals with NSCLC could be recruited to a strength training intervention, whether the specific exercise configuration including cluster sets was feasible, and if the intervention could be delivered in a hybrid manner including videoconferencing and in person in individuals’ homes.
- What are the key feasibility findings? Our results indicated that a hybrid delivery of a resistance training intervention for individuals with stages I and II non-small cell lung cancer is feasible, as indicated by our recruitment rates (*n* = 14 over 8 months), retention (79%), and intervention fidelity (83.1 ± 13.1%). Furthermore, > 90% of participants positively rated all aspects of the intervention delivery.
- What are the implications of the feasibility findings for the design of the main study? This intervention is feasible to administer in a larger trial, though several adaptations to the program for future interventions are required, particularly concerning the hybrid delivery approach (namely, the frequency, location and purpose of in-person visits, and utilization of breakout rooms for more tailored exercise instruction with group sessions).

## Background

Lung cancer is the second most diagnosed cancer in males and females, with over 200,000 new cases per year in the USA [1, 2]. Non-small cell lung cancer (NSCLC) is the prevailing subtype of lung cancer, accounting for ~ 85% of cases [3]. The 5-year survival rate for NSCLC is approximately 56% for localized tumors and 29% for regional tumors [2, 4]. Furthermore, individuals treated (e.g., surgery, radiation, chemotherapy) for NSCLC often have long-lasting psychological and physiological burden from the disease and its treatment (e.g., dyspnea (shortness of breath), pain, and fatigue) that contribute to physical disability, morbidity, and mortality [5–8].

Exercise training is commonly recommended in the management to cancer-related symptoms [9–11]. Specifically, resistance exercise may have unique benefits for muscle strength and physical function while attenuating muscle loss that is typically experienced in this patient population [7, 12–14]. However, symptom clusters following treatment completion (e.g., dyspnea and fatigue) have been recognized as contributors to the reduction in exercise capacity and functional decline in individuals with lung cancer [3, 5, 6, 15, 16]. It has also been suggested that the physiological and psychosocial burden of dyspnea may result in the avoidance of activity resulting in a cycle of deconditioning, exertional dyspnea at lower exercise intensities, followed by further deconditioning [13, 17, 18]. Consequently, symptom-related exercise avoidance in lung cancer accelerates deconditioning and the resultant trajectory towards physical disability, placing individuals at an even higher risk of disease and death [8, 18–21]. Given the debilitating effects of dyspnea in individuals with lung cancer, and the known influence of dyspnea on exercise capacity, there is a strong rationale to investigate strategies aimed at managing dyspnea during exercise in this population [15].

Managing dyspnea during exercise in individuals with high symptom burden is not unique [22, 23]. Interval cardiovascular training has been investigated to facilitate exercise at higher intensities/workloads while minimizing symptom burden such as dyspnea [24–27]. It is also possible to configure resistance exercise set prescription in an “interval”-like manner, by incorporating additional rest periods during each set of an exercise (i.e., cluster sets) [22, 24, 26, 28–31]. Strategically, incorporating extra rest during sets of resistance exercise may allow individuals with NSCLC to better tolerate exercise, which could result in greater improvements in physical function and quality of life [32–37].

Home-based-exercise interventions are rapidly emerging as a cost-effective way to overcome many of the traditional barriers to exercise interventions (travel, limited hours, resources, etc.). However, the delivery of exclusively home-/remote-based resistance exercise is notoriously challenging, particularly when attempting to find a safe place to exercise, adapting exercise to physical limitations, and selecting safe and appropriate exercises [38]. Consequently, we hypothesize that a hybrid approach (i.e., part remote, part in person supervised) can overcome many of these challenges, via the presence of an experienced staff member aiding the selection of safe and appropriate exercise and loading while also transitioning towards a hybrid approach that could foster autonomy and independence [39].

Though there is a strong rationale for the therapeutic effects of resistance exercise in NSCLC, conclusive evidence for its effectiveness is non-existent [3]. Thus, while the results of a recent meta-analysis indicate that recruitment (median = 59%; range = 9–100%) and retention (median = 86%; 50–100%) rates for exercise trials in lung cancer are somewhat similar to the field of exercise oncology, only one trial was exclusively using resistance exercise [40]. Consequently, little is known regarding the potential benefit and challenges of the hybrid delivery of resistance exercise in this population. Taken collectively, understanding the feasibility of conducting a hybrid delivery resistance training intervention for individuals with lung cancer is a critical first step prior to progression to a larger clinical trial assessing effectiveness of the intervention [41, 42].

The aim of our study was to examine the feasibility and acceptability of an 8-week hybrid delivery home-based resistance training (RT) program in individuals previously treated for NSCLC. A secondary aim of the study was to quantify changes in physical function (6-min walk test and sit-to-stand), muscular strength (5-repetition maximum for leg extension and chest press), and quality of life (Functional Assessment of Cancer Therapy-Fatigue and Dyspnea) in participants who completed the program.

## Methods/design

A protocol of our single-arm feasibility trial to investigate an 8-week hybrid delivery of RT with individuals treated for NSCLC stages I–III, including criteria for success and progression to a larger trial, has been previously published [39]. Assessments were completed at baseline and week 9. The study protocol was approved by the University of South Carolina’s Institutional Review Board (Pro00110261). All participants provided written informed consent prior to any study activity. The study was registered on ClinicalTrials.gov (NCT05014035). Success of the intervention and criteria to progress to a larger trial was based on the following:

1. Recruitment: The recruitment goal of *n* = 15 in 1 year has been reached. As mentioned previously, the sample size was determined based on gains in precision surrounding the mean and variance and ability to estimate parameters for future studies by recommendations from Julious et al. [43].
2. Retention: If ≥ 75% of the sample recruited to participate return for follow-up testing
3. Intervention fidelity: If relative dose intensity (RDI) (outlined below) is ≥ 70%

RDI was used to report intervention fidelity. Session volume was calculated as the product of the number of sets multiplied by the number of repetitions for each exercise and summed to give total volume for each session. The proportion of volume achieved relative to what was prescribed was used to give a RDI for each participant and then averaged to determine fidelity to the RT intervention [44].

### Changes to study protocol

Several changes to the study protocol were made since its initial design and publication of the protocol paper. Initially, we had planned to recruit individuals who had been treated for NSCLC within the previous 12 months. This was intended as a way to address a research gap where evidence for exercise in the phase immediately following the completion of treatment is lacking [9]. Individuals within this timeframe may still be recovering from the acute side effects of treatment, resulting in a greater treatment burden. As such, understanding if exercise can target symptoms during this timeframe could be valuable in better understanding how best to mitigate symptom burden [9]. Shortly after the initiation of recruitment, it was decided that there was not a strong enough rationale to exclude individuals more than 12 months post-treatment, and that their inclusion would allow for a larger recruiting pool and enhance generalizability. Furthermore, the use of registry data as a primary recruitment method resulted in a lag in obtaining information regarding participants being treated recently. As such, the change was made to remove the 12-month restriction on time since treatment completion and have an open-ended window on time since treatment completion. Secondly, the original eligibility criteria excluded those who were uncomfortable with study staff coming to their home for in-person sessions. However, upon discussion, the study team felt that this was unnecessarily excluding individuals. Consequently, anyone who was uncomfortable with house visits (as was the case with one participant in this trial) were offered the option of having the same sessions at the laboratory on campus.

Additionally, there were also staff shortages due to COVID-19 and other factors, as well as participant scheduling conflicts, which resulted in changes to the frequency of house visits during the initial 2-week in-home visits and a more flexible approach of visiting participant’s homes periodically to adjust/progress exercises (rather than on every sixth session) being adopted. Importantly, the change in the initial 2-week home visits resulted in this period being considered a pre-intervention familiarization period (to familiarize participants to the exercises, videoconference log in procedures, structure of the sessions, etc.), whereas the remaining 6 weeks were considered the full intervention period. Other modifications included the configuration of zoom sessions to include breakout rooms and incorporating campus visits for individuals not comfortable with study staff coming to their home. A summary of the original protocol, challenges presented, changes made, and rationale can be found in Supplementary Table 1.

### Participant recruitment and screening

Participants were recruited through collaboration with a local medical/radiation oncology private practice in Central South Carolina. Recruitment ran from November 2021 to June 2022. The primary source of recruitment was through the cancer registry databases. A waiver of Health Insurance Portability and Accountability Act authorization was approved by the institutional review board and the local hospital’s privacy board, to allow invitations signed by treating medical and radiation oncologists to be mailed to potential participants. Specifically, the intervention was conducted in waves of 3–6 participants. Therefore, to reduce the likelihood of losing participants due to time from initial contact to beginning the study, we opted to mail batches of invitations at the start of the study (for the first wave) and in the weeks following completion of the previous wave (in preparation for an upcoming wave). These invitations were “opt-out,” where individuals were provided contact information for study staff and were required to call if they did not want to be contacted regarding participation. A week after mailings were sent, study staff then called participants to discuss the study and its intended goals, risks, and benefits and to determine eligibility. Other methods of recruitment included flyers displayed in clinicians’ offices and word of mouth through healthcare providers.

#### Participants

Individuals were eligible to participate in the study if they had been diagnosed with NSCLC stages I–III, had completed all cancer treatments, and had no contraindications to performing RT in accordance with the American College of Sports Medicine’s guidelines on pre-exercise screening and contraindications to exercise training [45]. Medical clearance was required prior to participation in any study activities. Participants were also required to have stable Internet access and be able to read/understand English. Participants were excluded if they had a diagnosis of advanced (stage IV) lung cancer, due to concerns around the additional burden and high mortality rate in this advanced stage of disease. Furthermore, individuals were excluded if they had a diagnosis of small-cell lung cancer or were participating in structured *RT* ≥ 2 times/week for the past 6 months. There were no specific exclusion criteria set for individuals receiving medical treatment for diseases/conditions other than cancer.

#### Intervention

The intervention was delivered by trained study staff/graduate research assistants with experience of delivering exercise programs to individuals with cancer. All study staff delivering the exercise intervention were required to complete internal training in exercise prescription as it relates to individuals with cancer and were directly supervised by the principal investigator (CMF) who has extensive experience in delivering RT interventions for individuals with cancer. The intervention was designed to have a combination hybrid delivery of in-person home visits, and live, virtual sessions delivered via Zoom. The first 2 weeks involved home visits to determine a safe place to exercise in individuals’ homes and to provide in-person instruction on safe exercise, ensuring proper technique and appropriate loading for each exercise. Furthermore, the house visits involved setting up tablets that were provided to participants for Zoom sessions and ensuring participants could log in and follow instructions, and the tablets were positioned correctly to get a full-body view of participants to assist with exercise instruction. Participants were provided with dumbbells, steps, and suspension trainers, for the duration of the intervention. The goal of this 2-week period was to aim for 1–2 sets of ~ 12 repetitions (Table 2), with a specific focus on anchoring SD RIR to the loading based on participants perception, and study staff judgement of the movements/loading. However, as mentioned above, there was flexibility with strictly adhering to 1–2 sets of ~ 12 repetitions during these visits, with a focus on ensuring safe movements and comprehension with the scale anchoring and intervention procedures (i.e., zoom log in instructions and audio/video quality).

Following the 2-week home visit, the intervention was delivered via Zoom in small group sessions. Furthermore, two additional house visits were conducted across the 6 weeks of remotely delivered exercise, to work with participants in person and to modify exercises if necessary, to provide additional equipment, and/or to ensure movements were progressing safely, appropriately, and according to plan (Table 1).

**Table 1:** Overview of planned hybrid delivery approach

	**Weeks**																					
	**1**		**2**		**3**			**4**			**5**			**6**			**7**			**8**		
Video					✓	✓	✓	✓	✓		✓	✓	✓	✓	✓		✓	✓	✓	✓	✓	
In person	✓	✓	✓	✓						✓						✓						✓

The exercise sessions comprised of a warm-up, “main session” including six total movements targeting total body musculature (hip hinge, squat, horizontal push, vertical push, vertical pull, trunk) and a cool-down period. The initial set and repetition scheme (i.e., 8–12 repetitions) were informed by ACSM’s guidelines for resistance exercise prescription for individuals with cancer [9]. This program was piloted on 2 individuals’ representative of the participants fitting inclusion criteria of the trial. These participants underwent exercise sessions similar to what would be expected from those in the study and asked to provide feedback on various aspects of the exercise sessions (level of technicality, perceived level of difficulty, safety concerns, and potential challenges). Based on this feedback, components of the exercise sessions were modified prior to beginning the trial. Specifically, we had initially planned for a ~ 20-s rest every two repetitions for each movement. Feedback from these participants resulted in an adoption of ~ 15-s rest every 3–4 repetitions, to allow for more work to be completed prior to intra-set rest periods.

Online exercise sessions were delivered in a ratio of up to 4:1 from participant to instructor. Moreover, individual levels of physical function were observed during baseline testing by study staff. This information was then used to inform the selection of smaller “break-out” groups for virtual exercise sessions, based on level of function. These decisions were made at the discretion of the study staff involved in delivering the intervention. Sessions were progressed either by increasing load or changing body position/range of motion as was appropriate and tolerated. The overall structure of the planned progression is presented in Table 2. The loading was determined by “self-determined repetitions in reserve” (SD RIR). This was based on each individual’s perceived number of repetitions remaining following the completion of any given set. Participants were introduced to SD RIR (scale instructions and anchoring procedures) during the home visits in the familiarization phase. This was also an opportunity for study staff to help participants identify the appropriate load for a given RIR. The specific exercise selection and modifications to the program were tailored to each individual based on their movement patterns, pre-existing impairments, and/or study staff discretion. Further details on each participants’ exercise program are presented in the “Results” section.

**Table 2:** Planned progression of program

**Week**	**Sets**	**Total reps per sets**	**Set structure** Reps (rest in seconds)	**Loading (SD RIR)**
**1**	1–2	12	4 (15) 4 (15) 4	~ 3
**2**	1–2	12	4 (15) 4 (15) 4	~ 2
**3**	3	12	4 (15) 4 (15) 4	~ 2
**4**	3	10	4 (15) 3 (15) 3	~ 2
**5**	3	10	4 (15) 3 (15) 3	~ 4^a^
**6**	3	10	4 (15) 3 (15) 3	~ 2
**7**	3	8	4 (15) 4	~ 2
**8**	3	8	4 (15) 4	~ 2–3

*SD RIR* self-determined repetitions in reserve

^a^Week 5 had a focus on increasing the speed of muscular contractions with a focus on muscle power

### Study outcomes

Baseline and post-intervention data collection took place within 1 week of initiation and completion of the program at the University’s Department of Exercise Science (Table 3).

**Table 3:** Overview of testing and timeline of study activities

Outcomes	Baseline	Weeks 1–8	Post-testing
Feasibility: recruitment, retention, fidelity, acceptability	X	X	X
Health/wellness questionnaires: dyspnea, fatigue, quality of life	X		X
Muscular strength: 5 repetition maximum, 5 times sit to stand	X		X
Exercise capacity: 6-min walk test	X		X

### Feasibility and acceptability outcomes

The primary outcome of feasibility was evaluated by (1) recruitment (successful recruitment of target sample size, *n* = 15, in 1 year), (2) retention (≥ 75%), and (3) intervention fidelity (proportion of exercise completed, relative to what was prescribed, with ≥ 70% considered successful) [44].

### Secondary outcomes

#### Acceptability

Intervention acceptability was assessed using a 10-item questionnaire adapted from McDonnell et al., assessing the acceptability of intervention components on a 4-point Likert-type scale from “strongly disagree” to “strongly agree” [46]. Individuals were asked about the quality of audio/video for virtual sessions, level of difficulty in instructions and exercises, utility of home visits, and likelihood of participating in similar programs in the future.

#### Fatigue

Fatigue was assessed using the FACIT-Fatigue scale [47]. The FACIT-Fatigue scale evaluated how fatigue has impacted an individuals’ well-being in various ways (i.e., physical, social, emotional, functional) in the preceding 7 days. The scale is scored on Likert-type response from “not at all” (0) to “very much” (4). Lower scores indicate greater fatigue. The FACIT-F has extensive published evidence on its validity and reliability for individuals with cancer [48–51]. Cronbach’s alpha > 0.90 [50, 51].

#### Quality of life

The Functional Assessment of Cancer Therapy-Lung Cancer Subscale (FACT-LCS) was used to assess lung cancer-specific quality of life [52]. The LCS is a 9-item scale, with items scored on a 5-point Likert scale from 0 = “not at all” to 4 = “very much,” anchored to the past 7 days. Higher scores indicate better quality of life. The validity and reliability of the FACT-LCS have previously been demonstrated in lung cancer (coefficient alpha = 0.68–0.87) [52, 53].

#### Dyspnea

The FACIT-dyspnea (FACIT-D) 10-item short form was used to assess dyspnea [54]. The FACIT-D scores the level of dyspnea experienced with different activities (part 1) and the difficulty performing those activities because of dyspnea (part 2), in the past 7 days. The scale is scored on a Likert-type response from 0 = “no shortness of breath” to 3 = “severely short of breath” (part 1) or 0 = “no difficulty” to 3 = “much difficulty” (part 2). Higher scores indicate worse dyspnea. The FACIT-D has previously been demonstrated to be a valid and reliable measure of dyspnea in individuals with chronic obstructive pulmonary disease and dyspnea patients [54, 55]. Cronbach’s alpha = 0.973.

#### Muscular strength

A five-repetition maximum (5RM) test was used to assess both upper body (chest press) and lower body (leg extension) muscular strength [44, 56, 57]. Participants began each exercise with a general warm-up, followed by 2–3 exercise-specific warm-up sets (4–6 reps), gradually increasing load towards the approximate 5RM load. Once warm-up sets were completed, participants were then asked to complete a 5RM attempt (i.e., the amount of weight able to be lifted through full range of motion for a maximum of five repetitions without technique breakdown). If the individual was successful at completing 5 repetitions, the load was increased for a subsequent effort. Approximately, 3-min rest was provided between sets, with the last load completed safely recorded as the final 5RM score for each exercise. In an effort to minimize the impact of fatigue, every effort was made to achieve a 5RM in as few sets as possible [44, 56, 57]. Machine settings for each exercise were recorded and replicated for follow-up testing.

#### Physical function

The 6-min walk test (6MWT) and the 5 times sit-to-stand were used to assess physical function. The 6MWT consisted of two cones placed 30 m apart. Participants were asked to walk as far as possible at a brisk pace in a 6-min period. The total distance covered in 6 min was recorded as the final score. If participants needed to stop the test before the time ended, the final distance achieved before stopping was recorded as their final score. The 5 times sit-to-stand involved a chair (approximately 17-inch high, without arm rests) placed against a wall for stability and recording the time (in seconds) taken to stand 5 times from a sitting position as quickly as possible [58]. Arms were placed crossed on opposite shoulders, and a repetition was defined as moving from a seated position to full standing position. The same chair was used for each test [59].

#### Body composition

Body composition (whole body and appendicular lean mass, in addition to fat mass and bone mineral content) was assessed via dual-energy X-ray absorptiometry (DEXA) [60]. Participants were asked to attend the laboratory in a fasted state (at least 4 h) and refrain from drinking water for ~ 1 h before testing. Appendicular lean mass from DEXA and score on the 5 times sit-to-stand were be used to quantify the proportion of individuals who were sarcopenic at baseline [61]. Specifically, cutoffs for sarcopenia included low muscle quality using appendicular lean mass/height^2^ (< 7.0 kg/m^2^ in men and < 5.4 kg/m^2^ in women) and > 12 s on the 5 times sit-to-stand [61].

### Adverse events

Adverse events were recorded on a per-event basis and were recorded in accordance with the university’s standards, including a description of the event, its relation to the intervention (i.e., not related, unlikely, possibly, probably, definite), its level of seriousness (i.e., non-serious, required hospitalization, resulted in persistent disability, life-threatening, or resulted in death), and its intensity (i.e., mild, moderate, severe, life-threatening).

### Statistical analyses

Descriptive statistics were used to describe medical/demographic characteristics and feasibility and acceptability outcomes (e.g., recruitment, retention, fidelity, and acceptability). Due to the small sample size and lack of normal distribution for many of the secondary outcomes, median and interquartile range are reported for these outcomes at baseline and follow-up [43]. Median change score and interquartile range are presented from baseline and follow-up testing on a per-protocol basis. Deidentified data sets and analysis can be found at https://osf.io/6aktb/.

## Results

**Fig. 1:**
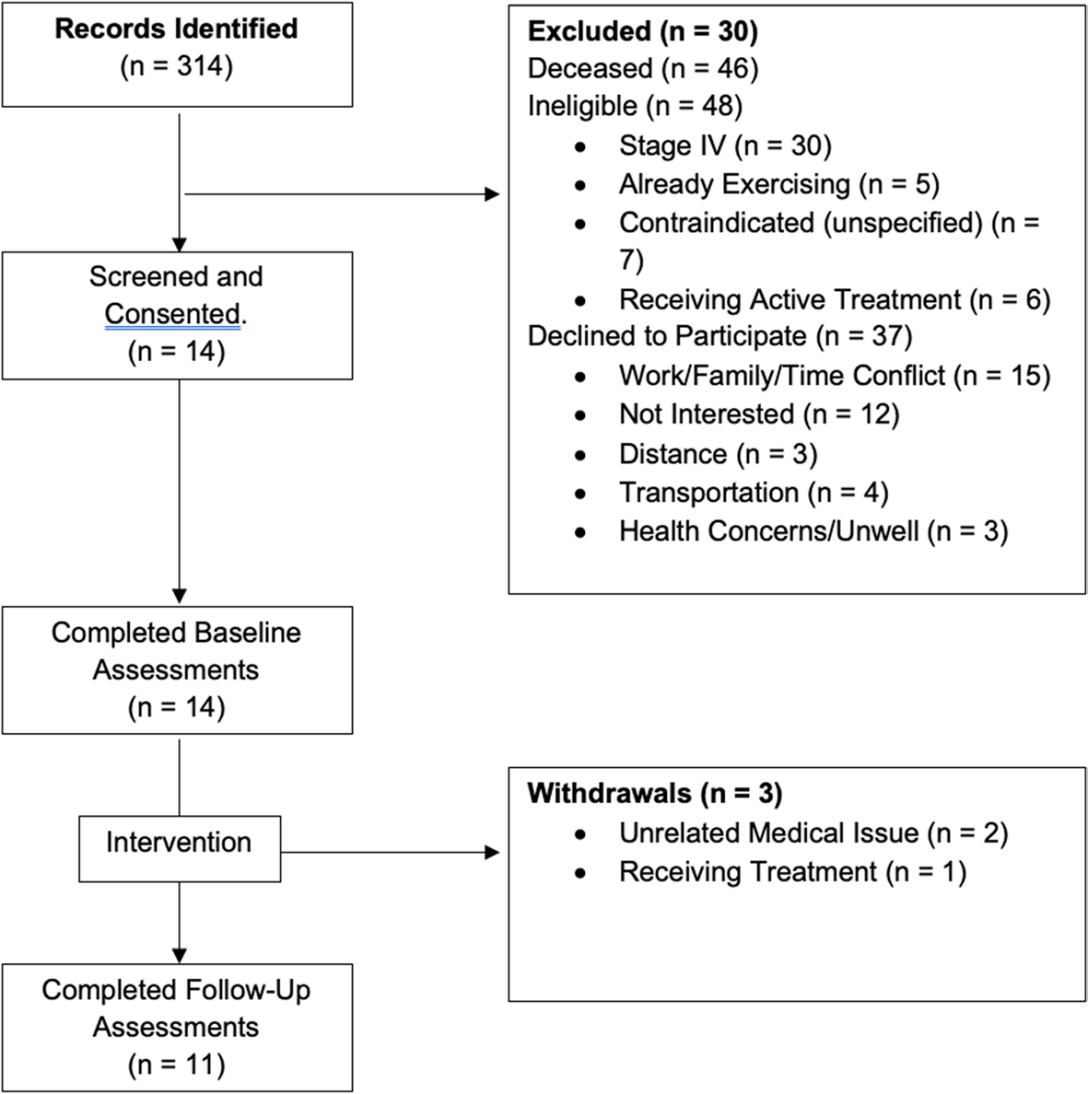
CONSORT diagram of participant flow through study [62]

Two batches of mailings were completed over a 6-month period, totaling 314 invitations. We were unable to contact 180 potential participants due to returned mail (with no forwarding address) or disconnected numbers. After returned addresses and/or disconnected numbers, 134 individuals received a follow-up phone call to discuss the trial and review eligibility criteria to determine if they were eligible for participation. After phone calls, 14 individuals (9.57%) were recruited to participate in the program. An overview of the recruitment and retention process can be found in Fig. 1. Of 14 participants recruited, 11 completed the intervention (2 withdrew due to unrelated illness (1 incidence of COVID (week 7), 1 exacerbation of chronic obstructive pulmonary disease (COPD) (week 6)), and 1 withdrew due to requiring active treatment), yielding a retention rate of 79%. There were no adverse events related to the intervention. Though we successfully collected secondary outcome data for the majority of individuals, several individuals did not complete some of the testing, leaving reduced numbers for these tests (i.e., 6MWT, *n* = 8; LE 5RM, *n* = 9; CP 5RM, *n* = 9). Primary reasons included preexisting injuries or discomfort using machines, in addition to time constraints regarding testing battery.

### Participant characteristics

Participants who completed the intervention (*n* = 11) were 27% male, 36% Black, with a mean age of 71 ± 10 years and mean BMI of 29.1 ± 6.5. Ten participants were stage I (91%), one was stage II (9%), and the average time since diagnosis was 62 ± 51 months. Select participant demographic and medical characteristics are presented in Table 4.

**Table 4:** Baseline participant characteristics

Characteristics	Total (*N* = 11) *n* (%) or m ± SD
**Biological Sex**	
Male	3 (27.3%)
Female	8 (72.7%)
**Age**	71.91 ± 9.87
**Height (in)**	66.81 ± 4.35
**Weight (lbs)**	187.73 ± 59.49
**Ethnicity**	
White, not of Hispanic origin	7 (63.6%)
Black/African American	4 (36.4%)
**Cancer stage**	
I	10 (90.9%)
II	1 (9.1%)
**Treatment characteristics**	
Surgery	5 (45.5%)
Surgery & chemotherapy	3 (27.3%)
Surgery & radiotherapy	1 (9.1%)
Radiotherapy	2 (18.2%)
Immunotherapy	0 (0%)
Time since diagnosis (months)	62 ± 51
**Sarcopenic**	2 (18.2%)
**Comorbidities (self-report)**	
Cardiovascular disease	1 (9.1%)
Respiratory conditions	7 (63.6%)
Diabetes	2 (18.2%)
Osteoporosis/osteoarthritis	2 (18.2%)

### Fidelity

Participant RDI and attendance, and reasons for missed/modified sessions, are presented in Table 5. Mean session attendance was 86.4 ± 9.5%. Mean intervention fidelity was 83.1 ± 13.1%. The primary reasons for missed sessions were illness and personal commitments (i.e., conflicting appointments, family emergencies). The primary reasons for modification of exercises were fatigue or time constraints (i.e., sessions were started late or had to be ended early). De-identified training logs including exercise selection, sets, reps, and load; full description of exercises prescribed to each individual, in addition to planned/achieved volume; and RDI can be found in supplemental file 2.

**Table 5:** Mean session attendance and relative dose intensity

	**Mean (SD)**			
**Attendance**	86.4 (9.5)			
**Relative dose intensity**	83.1 (13.1)			
	**No. of participants (n)**	**Percentage (%)**	**No. of sessions (*n*)**	**Percentage (%)**
**Missed sessions**				
General illness	5	45.45	11	5.56
Personal	7	63.64	18	9.09
Not recorded	2	18.18	2	1.01
**Dose modification**				
Sets	9	81.82	14	7.07
Weight	10	90.91	24	12.12
Exercise selection	7	63.64	8	4.04
Exercise removal	3	27.27	6	3.03
**Modification reason**				
Fatigue	10	90.91	25	12.63
Range of motion	1	9.09	1	0.51
Upper extremity pain	2	18.18	3	1.52
Lower extremity pain	5	45.45	11	5.56
Other (i.e., time constraints)	8	72.73	13	6.57

### Acceptability

With regard to acceptability, > 90% of participants positively rated (i.e., selected either agree or strongly agree on the acceptability questionnaire) all aspects of the intervention delivery (i.e., ease and quality of virtual delivery, level of difficulty, and home-based approach). Individual components of the questionnaire are presented in Table 6.

**Table 6:** Responses to acceptability questions

Acceptability statements	Positive responses (*N* = 11)	Strongly disagree	Disagree	Agree	Strongly agree
It was easy to use the tablet to log on for exercise sessions	11 (100%)			4 (36%)	7 (64%)
The audio quality for virtual exercise sessions was good	10 (90.9%)		1 (9%)	6 (55%)	4 (36%)
The video quality for virtual exercise sessions was good	11 (100%)			4 (36%)	7 (64%)
It was easy for me to record what I did for each session	8 (72.7%)		3 (27%)	5 (45%)	3 (27%)
The level of difficulty was just right for me	10 (90.9%)			7 (63%)	3 (27%)
Having a home-based setup worked well for me	11 (100%)			4 (36%)	7 (64%)
The exercise instructions were clear	11 (100%)			4 (36%)	7 (64%)
I would participate in a similar program in the future	11 (100%)			3 (27%)	8 (83%)
Having home visits throughout the program was helpful^a^	10 (90.9%)			5 (45%)	5 (45%)
The exercise equipment was easy to use	11 (100%)			4 (36%)	7 (64%)

^a^One individual did not respond as they opted for campus visits rather than home visits

Means and standard deviations for fatigue, quality of life, dyspnea, 5RM, 6MWT, and 5 times sit-to-stand are presented in supplementary file 3. The small sample size and lack of control group preclude any comprehensive analysis on changes in these outcomes. However, standard cutoffs, or reference values of minimally clinically important differences (MCID) from similar populations (i.e., COPD or older adults), were used to interpret the changes in scores, where possible [63–70]. Following the intervention, participants experience meaningful improvements in 6MWT (median change = 38.05 m). No other outcomes changed meaningfully following the intervention.

## Discussion

The primary aim of this trial was to assess the feasibility of the hybrid delivery of a symptom-tailored resistance exercise program for individuals previously treated for NSCLC. Our recruitment rate (*n* = 14 in 8 months), retention (79%), and exercise fidelity (83.1 ± 13.1%) met the majority of our predefined indicators of feasibility. Of note, we originally planned to recruit *n* = 15 to this study. However, the study experienced significant delays as a result of institutional review board processing and research staff changeover. Consequently, the study was stopped approximately 8 months after recruitment efforts began as opposed to 12 months, due to funding and resource constraints. However, we are confident that recruiting *n* = 14 in 8 months is reflective of our prior goal of recruiting *n* = 15 in 1 year.

Our study was open to individuals with stages I–III NSCLC. All participants were either stage I (91%) or stage II (9%), and time since diagnosis was ~ 62 ± 51 months. We primarily used cancer registry data to identify potential participants, mailing invitations to individuals’ homes. The survival rate for lung cancer decreases exponentially for lung and bronchus cancer at more advanced stages of disease (5-year survival: ~ 60% localized; ~ 30% regional spread; ~ 6% distant spread) [1]. Consequently, identifying individuals with stage III NSCLC through cancer registries may result in challenges to recruitment due to other priorities, poor health, and/or mortality. Our results are also similar to other exercise trials in lung cancer, recruiting a significantly higher proportion of stage I/II NSCLC, relatively to more advanced stages [71]. Consequently, recruiting individuals with stage III NSCLC to exercise trials may need more active recruitment strategies, including direct referrals from oncologic team, and/or recruiting early in the cancer continuum. We recruited 14 individuals from a total of 134 individuals contacted, representing a recruitment rate of 9.57%. This is somewhat lower than the median of ~ 38% that has been previously reported in exercise oncology trials [72]. Of those contacted, *n* = 48 (36%) were ineligible (stage IV; receiving active cancer treatment, contraindicated), and *n* = 37 (28%) declined (not interested, too busy/personal commitments). Reasons for declining to participate were common and align with other exercise oncology trials [72]. Though we view our recruitment as successful, the challenges experienced during recruitment, and relatively low recruitment rate, warrant consideration for future RCT’s, where multi-site trials might be warranted to increase the potential pool of participants.

We reached our retention target of > 75%, with a retention rate of 79% in this study. One participant withdrew due to active treatment status rendering them ineligible for the trial. Of the remaining participants, one withdrew in the last week of the study due to COVID-19 and exacerbation of COPD. The other participant withdrew because of progression of respiratory symptoms resulting from comorbidities. Despite this, our retention rate is in line with other exercise oncology trials in lung cancer, with an anticipated drop out typically estimated at ~ 20% [40]. The exercise fidelity of 83% also compares favorably to trials of exercise in lung cancer [14, 40, 73]. Notably, it is recognized that individuals with lung cancer may have a higher disease/symptom burden relative to common cancers such as breast and prostate and have a more difficult time adhering to exercise interventions [14, 74]. The high exercise fidelity observed in our study could be a result of our individualized approach, using home visits to tailor exercise prescription, using cluster sets to mitigate fatigue/dyspnea and high level of engagement on Zoom and in person, or a combination of all the above. This is in line with prior studies of exercise and lung cancer, with attendance rates ranging from 44 to 100% [40]. Nevertheless, the recruitment, retention, and fidelity rates observed in our trial, in addition to the positive responses to questions regarding acceptability, support the feasibility of our intervention and the progression to the next phase of research (e.g., phase II clinical trial to investigate the impact of the intervention on key outcomes such as physical function and dyspnea).

Though our results support the feasibility of hybrid approach to delivering cluster-set RT in NSCLC, our trial was not without challenges, and several modifications to our pre-planned protocol were implemented. Notable changes were made to the intervention delivery. Namely, it was originally planned to have an initial 2-week period of home visits to ensure safe set up of exercise stations, selection of appropriate exercises/loading, and familiarity with remote set up. Secondly, it was planned that study staff would go out to participant homes every 6th exercise session to provide feedback on exercise technique and ensure progression of exercises/loading. However, we recruited participants from a ~ 30-mile radius of the study site. Consequently, significant challenges (i.e., travel requirements of study staff, scheduling multiple participants during the same days/weeks) were experienced in scheduling participants initial home visits and subsequent check-in visits.

Importantly, however, the home visits were considered a valuable component of the intervention. In response to the acceptability questionnaire, all participants agreed that having a home-based setup worked well for them, and all participants who had home visits reported that having home visits throughout the program was helpful. This is reflective of other exercise oncology trials delivering telehealth interventions that recommend some element of in-person contact to increase participant engagement and enhance support, particularly with exercise guidance and technology use [75]. Consequently, though the home visits added value to our remote approach, future research should look to investigate the most efficient and cost-effective approach of incorporating home visits or in-person contact points into remote resistance exercise interventions. For example, future trials could look to partner with community centers or other sites that could be grouped geographically. This way, participants could meet study staff at common sites for in-person visits and familiarization, rather than individual house visits, which could alleviate the burden on study staff.

A secondary aim of our study was to assess changes in physical function, muscle strength, dyspnea, and fatigue. We were able to successfully obtain data for these measures in the majority of participants. However, participants described difficulty with 5RM testing, due to preexisting injuries and/or discomfort with machines used for testing. Given the lack of meaningful change observed in 5RM values, the time taken to administer these tests, and the reports of discomfort by some participants, obtaining objective measures of strength may require changes in the selection of tests (e.g., isometric tests using a dynamometer) or using machines or movements (e.g., seated leg press, with more room to accommodate individuals with limited mobility) that might be more suited to this patient population. There was also no meaningful change in quality of life, or dyspnea, for participants who completed the intervention. Participants had relatively high baseline levels of quality and life and low levels of dyspnea, which could have impacted the ability to observe positive changes. Furthermore, though meaningful improvements in 6MWT were observed, the small sample size, lack of control group, and wide variability in changes preclude any meaningful interpretation. We have demonstrated the feasibility of obtaining these measures in individuals with NSCLC, supporting their inclusion in a future trial. However, the reduced number of participants with complete data for all secondary outcomes highlight the potential burden of the test battery. Consequently, the inclusion of all these outcomes in a future RCT would require careful consideration of their value relative to burden/time requirement.

A strength of this study is the defined, a priori benchmarks of feasibility that were established and mostly achieved. Another important strength of this study was the diverse gender and racial profile of the sample. We understand that many social and cultural factors influence the participation of minorities in clinical trials. We credit our primary recruitment method with this diverse profile [46]. Furthermore, the hybrid delivery of an exercise intervention enabled some in-person sessions with study staff which helped ensure the safety and comfort of participants, while having the majority of the intervention being delivered remotely to overcome typical barriers to supervised exercise interventions (i.e., travel and time constraints). However, our study has several limitations. Firstly, we did not use cut points of dyspnea to specifically enroll individuals with dyspnea after treatment for NSCLC. This likely impacted the variability of dyspnea levels observed across participants and the likelihood that changes would be observed. However, patient-reported dyspnea is difficult to measure and has challenges such as the inability to recall baseline measures, changes in perception of dyspnea over time, and lifestyle adjustments to avoid/reduce dyspnea. These factors make it difficult to accurately quantify the burden of dyspnea. It is also possible that a lifestyle intervention including exercise influenced the recruiting of earlier stage disease and/or those with lower symptom burden. As a first step in this line of inquiry, we were more interested in understanding recruitment and retention rates to a RT intervention in NSCLC. Lastly, anecdotal feedback from participants revealed that the majority enjoyed the cluster-set approach as a way of making the sessions more tolerable. Future research may look to specifically include individuals with documented dyspnea to understand how resistance exercise could be employed in this population. The small sample size, lack of advanced stages of disease, and lack of control group limit the conclusions that can be drawn and generalizability of our results (i.e., to the large cohort of stages III and IV NSCLC). Furthermore, our study excluded individuals without stable Internet access. Consequently, it is worth exploring different models of home-based resistance exercise training (i.e., pre-recorded videos on tablets, telephone-guided exercise) to enhance the accessibility of these interventions.

## Conclusions

The hybrid delivery of a home-based resistance exercise program for individuals previously treated for NSCLC was found to be feasible and acceptable. Furthermore, no adverse events related to the intervention were reported, supporting the safety of this type of intervention. Adaptations to the program for future interventions are required, particularly concerning the hybrid delivery approach (namely, the frequency, location and purpose of in-person visits, and utilization of breakout rooms for more tailored exercise instruction with group sessions). Having established the feasibility of this intervention and identified appropriate adaptations to its components, a larger trial is warranted to test the effectiveness of the hybrid delivery of cluster-set RT in individuals with NSCLC. However, this trial design had a relatively high burden in terms of resources, personnel, and potential cost. Consequently, this burden should be weighted in the design of a future RCT.

### Supplementary Information

**Additional file 1: Supplementary Table 1.** Changes to Study Protocol

**Additional file 2.** De-identified training logs, full description of exercises prescribed to each individual, and RDI.

**Additional file 3.** Means and standard deviations for fatigue, quality of life, dyspnea, 5RM, 6MWT, and 5 times sit-to-stand.

## Data Availability

Deidentified data sets and analysis can be found at https://osf.io/6aktb/.
