# Supplementary material for: Hybrid delivery of cluster-set resistance training for individuals previously treated for lung cancer: the results of a single-arm feasibility trial
Source: Pilot Feasibility Stud. 2023 Oct 17;9:177. doi: 10.1186/s40814-023-01405-z (PMC10580552; doi:10.1186/s40814-023-01405-z)
Supplement: Supplementary file 1 — Additional file 1: Supplementary Table 1. Changes to Study Protocol [file 40814_2023_1405_MOESM1_ESM.docx]

Supplementary Table 1. Changes to Study Protocol

| **Original protocol** | **Challenge(s)** | **Change made** | **Rationale** |
| --- | --- | --- | --- |
| "The first two weeks will include a member of the study team visiting the home of participants (or other mutually agreed upon private location) two times per week to deliver supervised exercise sessions." | COVID-19 related scheduling issues; short staffed during summer months | Shifted to a two-week run-in period consisting first of a week of two in-person house visits for equipment delivery and in-person coaching; followed by two one-on-one zoom sessions to iron out any technical issues and emphasize form. | Changing the run-in period structure cut the number of initial house visits for each participant to two, removing travel time for study staff, while still allowing adequate time for in-person coaching and equipment drop off. The addition of one-on-one zooms also helped remove technical issues prior to transitioning to group session. |
|  | Certain participants did not feel comfortable with house-visits | Had orientation day on campus in lab and took equipment back home; followed by one-on-one zoom sessions to familiarize with movements | An orientation day was added to still allow for in-person coaching to introduce each movement and make any necessary adjustments to the exercise program based on the participant, both of which can be difficult over Zoom. |
| "Every sixth session, the exercise session will be delivered in-person to address any concerns, provide modifications if necessary, and ensure safe progression of exercises." | Scheduling issues on both sides (participant availability/study staff travel times) | Two house visits were scheduled and utilized for each participant during the 6-weeks of remotely-delivered exercise, while loosely scheduled around three-week periods, specific days were chosen based upon each participant's individual needs and progress. | Allowed for a more flexible implementation of the house-visits throughout the 6-week period; if progression in weight or changes in form were seen to be necessary a house-visit could be scheduled, allowing for an in-person check-in. |
| “Completed definitive treatment for localized NSCLC (stages I–III) within 12 months of completion” | The registry data being used to recruit had a lag time in being updated, which meant it was difficult to identify individual who had completed treatment in the past 12-months. Further, the study team | We changed the eligibility to include individuals who had completed primary treatments at any point, with no upper limit on time since treatment completion. | Allowed for a greater pool of participants to recruit from. Further, the rationale for excluding individuals greater than 12-months from treatment was not sufficiently strong, given the variability in symptom burden that can exist in the time following treatment completion. |
| "These sessions will be delivered in a ratio of up to 4:1 of participants to instructor." | Zoom communication issues when coaching a group; different levels of physical function across participants led to pacing issues | Utilization of breakout rooms (3 and 3) - separation based loosely on physical function. 2 head trainers (one for each room) 2 assistants/demonstrators (one for each room) | Breakout rooms allowed for more productive communication and coaching |
|  |  |  |  |
|  |  |  |  |
