# Supplementary material for: Hybrid delivery of cluster-set resistance training for individuals previously treated for lung cancer: the results of a single-arm feasibility trial
Source: Pilot Feasibility Stud. 2023 Oct 17;9:177. doi: 10.1186/s40814-023-01405-z (PMC10580552; doi:10.1186/s40814-023-01405-z)
Supplement: Supplementary file 3 — Additional file 3. Means and standard deviations for fatigue, quality of life, dyspnea, 5RM, 6MWT, and 5 times sit-to-stand. [file 40814_2023_1405_MOESM3_ESM.docx]

|  | Baseline | | Follow Up | | Baseline to Post Intervention | |  |
| --- | --- | --- | --- | --- | --- | --- | --- |
|  | Median | IQR | Median | IQR | Median | IQR (25^th^, 75^th^) | MCID* |
| **Physical Function and Strength** | | | | | | | |
| Gait Speed (m/s) (n = 11) | 1.04 | 0.30 | 1.02 | 0.17 | 0.03 | -0.05, 0.046 | 0.10 to 0.20 m/s^1,2^ |
| Sit to Stand (n = 11) | 14.34 | 5.15 | 12.41 | 1.65 | -1.26 | -3.93,-0.61 | 1.7 to 2.5s^3-5^ |
| 6MWT (n = 8) | 337.5 | 217.25 | 358.75 | 139 | 38.05 | -27.00, 83.50 | 22-32m^6^ |
| LE 5RM (n = 9) | 50.00 | 70 | 75.00 | 47.5 | 0.00 | -10.00, 10.00 | n/a |
| CP 5RM (n = 9) | 50.00 | 60 | 60.00 | 45 | -10.00 | -10.00, 10.00 | n/a |
| **Quality of Life** | | | | | | | |
| FACIT Dyspnea (level) (n = 9) | 23.00 | 18.00 | 20.50 | 16.5 | -3.0 | -4.00, -1.00 | n/a |
| FACIT Dyspnea (difficulty) (n = 10) | 18.00 | 13.75 | 15.00 | 8.00 | -2.5 | -3.75, 1.00 | n/a |
| FACIT-Fatigue (n =10) | 43.00 | 14.25 | 40.00 | 11.5 | 3.0 | -2.25, 7.25 | 4-6^7,8^ |
| FACT-LCS (n = 10) | 19.00 | 5.25 | 19.50 | 6.00 | 1.5 | 0.25, 3.00 | 2-3^9,10^ |

6MWT = six minute walk test; CP 5RM = chest press five repetition maximum; FACIT = Functional Assessment of Chronic Illness Therapy; IQR = Interquartile range; LE 5RM = leg extension five repetition maximum; MCID = minimally clinical important difference. *MCID reference populations: 1 = “adults with pathology” (stroke, hip fracture, multiple sclerosis); 2 = Chronic Obtrusive Pulmonary Disease; 3 = Stroke; 4 = Older females; 5 = Chronic Obtrusive Pulmonary Disease; 6 = Lung cancer; 7 = Cancer, Rheumatoid Arthritis or Systemic Lupus Erythematosus; 8 = Cancer; 9 = Lung Cancer; 10 = Non-Small Cell Lung Cancer.

**References**

1. Bohannon, R.W. & Glenney, S.S. Minimal clinically important difference for change in comfortable gait speed of adults with pathology: a systematic review. *Journal of Evaluation in Clinical Practice* **20**, 295-300 (2014).

2. Kon, S.S.C.*, et al.* The 4-metre gait speed in COPD: responsiveness and minimal clinically important difference. *European Respiratory Journal* **43**, 1298-1305 (2014).

3. Agustín, R.M.-S., Crisostomo, M.J., Sánchez-Martínez, M.P. & Medina-Mirapeix, F. Responsiveness and Minimal Clinically Important Difference of the Five Times Sit-to-Stand Test in Patients with Stroke. *International Journal of Environmental Research and Public Health* **18**, 2314 (2021).

4. Goldberg, A., Chavis, M., Watkins, J. & Wilson, T. The five-times-sit-to-stand test: validity, reliability and detectable change in older females. *Aging Clinical and Experimental Research* **24**, 339-344 (2012).

5. Jones, S.E.*, et al.* The five-repetition sit-to-stand test as a functional outcome measure in COPD. *Thorax* **68**, 1015-1020 (2013).

6. Granger, C.L., Holland, A.E., Gordon, I.R. & Denehy, L. Minimal important difference of the 6-minute walk distance in lung cancer. *Chronic Respiratory Disease* **12**, 146-154 (2015).

7. Nordin, Å., Taft, C., Lundgren-Nilsson, Å. & Dencker, A. Minimal important differences for fatigue patient reported outcome measures—a systematic review. *BMC Medical Research Methodology* **16**(2016).

8. Yost, K.J. & Eton, D.T. Combining distribution- and anchor-based approaches to determine minimally important differences: the FACIT experience. *Eval Health Prof* **28**, 172-191 (2005).

9. Cella, D.*, et al.* What is a clinically meaningful change on the Functional Assessment of Cancer Therapy-Lung (FACT-L) Questionnaire? Results from Eastern Cooperative Oncology Group (ECOG) Study 5592. *J Clin Epidemiol* **55**, 285-295 (2002).

10. Cella, D.*, et al.* Clinically Meaningful Improvement in Symptoms and Quality of Life for Patients With Non-Small-Cell Lung Cancer Receiving Gefitinib in a Randomized Controlled Trial. *Journal of Clinical Oncology* **23**, 2946-2954 (2005).
